# Gene expression profiles associated with aging and mortality in humans

**DOI:** 10.1111/j.1474-9726.2009.00467.x

**Published:** 2009-06

**Authors:** Richard A Kerber, Elizabeth O’Brien, Richard M Cawthon

**Affiliations:** 1 Department of Oncological Sciences2000 Circle of Hope, Salt Lake City, UT 84112-5550, USA; 2 Huntsman Cancer Institute, University of Utah2000 Circle of Hope, Salt Lake City, UT 84112-5550, USA; 3 Department of Human Genetics, University of Utah15 North 2030 East, Salt Lake City, UT 84112, USA

**Keywords:** aging, cell cycle, cell lines, CEU, gene expression, longevity

## Abstract

We investigated the hypothesis that gene expression profiles in cultured cell lines from adults, aged 57–97 years, contain information about the biological age and potential longevity of the donors. We studied 104 unrelated grandparents from 31 Utah CEU (Centre d’Etude du Polymorphisme Humain – Utah) families, for whom lymphoblastoid cell lines were established in the 1980s. Combining publicly available gene expression data from these cell lines, and survival data from the Utah Population Database, we tested the relationship between expression of 2151 always-expressed genes, age, and survival of the donors. Approximately 16% of 2151 expression levels were associated with donor age: 10% decreased in expression with age, and 6% increased with age. Cell division cycle 42 (CDC42) and CORO1A exhibited strong associations both with age at draw and survival after draw (multiple comparisons-adjusted Monte Carlo *P*-value < 0.05). In general, gene expressions that increased with age were associated with increased mortality. Gene expressions that decreased with age were generally associated with reduced mortality. A multivariate estimate of biological age modeled from expression data was dominated by CDC42 expression, and was a significant predictor of survival after blood draw. A multivariate model of survival as a function of gene expression was dominated by CORO1A expression. This model accounted for approximately 23% of the variation in survival among the CEU grandparents. Some expression levels were negligibly associated with age in this cross-sectional dataset, but strongly associated with inter-individual differences in survival. These observations may lead to new insights regarding the genetic contribution to exceptional longevity.

## Introduction

Systematic genome-wide screens for engineered gene expression changes that increase lifespan have identified scores of ‘longevity genes’, by deletion in yeast (Powers *et al.*, 2006), RNA interference in *Caenorhabditis elegans* (Hamilton *et al.*, 2005), and activation of expression in *Drosophila* (Seong *et al.*, 2001). Therefore, we hypothesized that natural inter-individual variation in gene expression in human populations may be associated with significant differences in survival. We investigated the possibility that shifts in expression levels of single genes (or combinations of genes) may have broad health benefits in late middle and old age, by lowering the risk of mortality. Such effects would be expected for genes that regulate the rate of progression of fundamental processes of senescence.

The CEPH/Utah (CEU) family resource, maintained at the Coriell Cell Repositories in Camden, NJ, USA (http://ccr.coriell.org/Sections/Collections/NIGMS/CEPHFamilies.aspx?PgId=49&Coll=GM), consists of Epstein–Barr virus-immortalized lymphoblastoid cell lines and DNA extracted there from, for 46 three-generation CEU families, each consisting of 5–15 siblings, their two parents, and two to four grandparents who were still alive at the time of the family blood draws in the early 1980s (White *et al.*, 1985; Dausset *et al.*, 1990). Cell lines from 153 CEU grandparents are available from Coriell. In addition, approximately 20 years of follow-up data (survival and mortality) are available for 145 of the 153 Utah grandparents from the Utah Population Database (UPDB) and the Utah Genetic Reference Project at the University of Utah.

Several recent papers examining the genetics of gene expression levels in the CEU cell lines (Cheung & Spielman, 2002; Cheung *et al.*, 2003, 2005; Schadt *et al.*, 2003; Monks *et al.*, 2004; Morley *et al.*, 2004; Stranger *et al.*, 2005) have confirmed, by familial correlation, linkage, and/or genetic association, that a substantial portion of the variability in gene expression levels is under genetic control. Thus, prior evidence has established that a major source of variation in expression levels in the CEU cell lines is genetic differences among individuals, rather than variation in the conditions of tissue culture, cell transformation, or storage and handling of the samples. In this context, it is reasonable to test for associations between gene expression profiles in these cell lines and genetically influenced complex traits in their donors.

To date, two genome-wide studies of longevity in humans have been published (Puca *et al.*, 2001; Lunetta *et al.*, 2007), and several additional studies are currently in progress. Puca and colleagues performed a sib-pair linkage study of centenarians and near centenarians, and identified a region of interest on chromosome 4q25. Subsequent work by this group led to the conclusion that one or more variants of microsomal triglyceride transfer protein (Geesaman *et al.*, 2003) were responsible for this effect, although other groups have failed to confirm this association (Nebel *et al.*, 2005; Beekman *et al.*, 2006). More recently, the first genome-wide association study of longevity and correlates of longevity found numerous associations with nominal *P*-values of 0.001 or less, including some candidate genes such as FOX1α, but none that clearly exceeded a threshold allowing for multiple hypothesis testing (Lunetta *et al.*, 2007).

In this paper, we report on the association between age at blood draw and expression levels for 2151 always-expressed genes in the CEU cell lines. We use these data to construct an estimate of biological age based on gene expression levels, then use our biological age estimate in a proportional hazards model of survival after blood draw to assess the degree to which gene expression profiles can serve as biomarkers of aging and/or longevity. We further test how well a multivariate survival model based on age-adjusted gene expression predicts mortality among the CEU grandparents. Our approach is not specifically designed to identify heritable variants that affect longevity. Rather, we are searching for stable variation in gene expressions that affect or mark longevity. We anticipate that inherited genetic variants, including copy number variants, account for much of the variation in gene expression, although it is likely that epigenetic factors may also play a large role (Mathers, 2006).

## Results

### Changes in gene expression with age: CEU grandparents

If the expression of a gene responds to the progress of senescence by rising or falling over the adult lifespan, then expression differences among chronologically age-matched adults may reflect variation in rates of biological aging. To establish age-related changes in gene expression levels, we first identified expressions most strongly associated with age at blood draw. Of the 8793 total measured expressions (probesets on Affymetrix HG-Focus arrays), we used only the 2151 always-expressed genes (in all 362 cell lines and three generations of Utah CEU families), to examine the relationship between individual expression levels and age at draw in the CEU grandparent cell lines. We modeled expression as a simple linear function of age at draw. Expression levels are reported on a log_2_ scale, so that a linear increase or decrease in measured expression level corresponds to a multiplicative increase in gene expression. Because the grandparents were effectively unrelated to one another, no adjustment for kinship was necessary, and conventional linear regression models were used.

A more complex analysis of changes in gene expression with age over all three generations of CEU families revealed a large number of highly significant associations. However, interpretation of age-related changes in expression across three generations, ages 5–97, is difficult because senescence can be confounded with sexual or physiological maturation, as well as secular trends occurring over such a long span of donor birth years in these families. Methods and results of this analysis are described in the Supporting Information.

Of the 2151 always-expressed genes, 345 (16%) expressions were associated with age at draw in the CEU grandparents at a nominal *P*< 0.05. Of these, 125 increased with age and 220 decreased with age. Table 1 shows the magnitude, direction, and significance of the linear regression of expression as a function of age at draw for the top ten age-associated expression levels for the 104 CEU grandparents, after adjusting for sex. None of the always-expressed genes was linearly associated with age at draw at a nominal *P*-value below the Bonferroni 5% threshold of 2.3 × 10^−5^. The strongest association of expression level with age was CDC42 (cell division cycle 42), which exhibited strongly increased expression with age, with a sex-adjusted *P*-value of 3.1 × 10^−5^, and an unadjusted *P*-value of 1.3 × 10^−5^. Among the ten most strongly age-associated expression levels (Table 1), equal numbers (five) increase and decrease with age. TableS3 (Supporting Information) shows complete results for the 2151 always-expressed genes.

**Table 1:** Top ten age-associated expression levels in CEU grandparents

HG-focus probeset	Gene symbol	*Z*	*P*-value	*H*^2^	Spouse correlation
HF6524	CDC42	4.36	3.14E−05	0.26	0.062
HF2432	MKNK2	4.09	8.65E−05	0.35	0.12
HF8737	SH3BGRL	4.07	9.51E−05	0.45	−0.057
HF4113	RNH1	−4.03	1.09E−04	0.00	−0.010
HF6098	TMEM142C	3.75	2.97E−04	0.09	−0.13
HF7682	CDC6	−3.73	3.13E−04	0.33	0.28
HF1646	USP1	−3.65	4.23E−04	0.23	0.055
HF1405	EDF1	3.60	4.88E−04	0.27	−0.028
HF982	QDPR	−3.60	5.01E−04	0.28	0.029
HF7873	CORO1A	−3.49	7.19E−04	0.66	0.23

Probeset, probeset name from HG-focus refseq transcript library [see reference (Liu *et al.*, 2007)]; gene symbol(s), HUGO symbol name or names corresponding to current mapping of probeset sequence; *Z*, *Z*-score of linear model of expression vs. age, adjusted for sex; *P*-value, probability of observing a *Z* greater than that observed under the null hypothesis; *H*^2^, heritability.

Also shown in Table 1 is the estimated heritability of expression (*H*^2^) for each gene listed, and the correlation of expression between spouses. Most of the genes listed in Table 1 have heritabilities between 0.2 and 0.5. CORO1A has the highest estimated heritability (0.66), while expression levels for RNH1 and TMEM142C do not appear to be heritable. Spouse correlations are generally very low, with moderate positive correlations observed for CDC6 (0.28) and CORO1A (0.23).

### Proportional hazards models of survival vs. gene expression

We also tested for gene expressions most strongly associated with survival, independently of their associations with age at blood draw. Table 2 shows the ten strongest associations between age-adjusted expression and survival after blood draw among the 104 CEU grandparents. Corresponding data for all genes are given in TableS3 (Supporting Information). None of the observed effects exceeds the Bonferroni threshold of 2.3 × 10^−5^, although CORO1A (coronin) comes quite close. Nine of the ten strongest associations of age-adjusted expression with mortality are negative, meaning that relative overexpression of the gene is associated with reduced mortality. Interestingly, the one exception is TERF2IP (telomeric repeat binding factor 2, interacting protein), which is thought to protect telomeric DNA from nonhomologous end-joining. Note that only one gene (CORO1A) appears in both Tables 1 and 2, supporting the notion that gene expressions strongly associated with age at blood draw are not necessarily strongly associated with survival, too. As in Table 1, most of the estimated heritabilities in Table 2 are 0.25 or greater, while CORO1A is again highest (0.66). No evidence for heritability of expression was seen for TERF2IP, KIF2C, or EMP3. We observed moderately strong positive spouse correlations for IQGAP1 (0.22) and CORO1A (0.23).

**Table 2:** Top 10 survival-associated expression levels in CEU grandparents

HG-focus probeset	Gene symbol	*Z*	*P*-value	*H*^2^	Spouse correlation
HF7873	CORO1A	−4.20	2.64E−05	0.66	0.23
HF8664	IQGAP1	−3.60	3.19E−04	0.59	0.22
HF6054	AURKB	−3.58	3.41E−04	0.34	−0.04
HF7038	TERF2IP	3.37	7.45E−04	0.064	0.0089
HF6482	CBX5	−3.37	7.46E−04	0.41	−0.058
HF8349	KIF2C	−3.35	8.02E−04	0.046	0.035
HF657	ACTR2	−3.27	1.07E−03	0.45	0.068
HF2735	SPAG5	−3.21	1.31E−03	0.39	−0.012
HF2854	MTF2	−3.17	1.54E−03	0.25	0.040
HF7574	EMP3	−3.13	1.74E−03	0.068	−0.0026

Probeset, probeset name from HG-focus refseq transcript library [see reference (Liu *et al.*, 2007)]; gene symbol(s), HUGO symbol name or names corresponding to current mapping of probeset sequence; *Z*, *Z*-score from proportional hazards model of survival vs. age-adjusted expression; *P*-value, probability of observing a *Z* greater than that observed; *H*^2^, heritability.

A total of 167 (7.8%) expression levels were associated with survival in the CEU grandparents at a nominal *P*< 0.05. Of these, 48 were associated with age at blood draw at a nominal *P*< 0.05. TableS2 (Supporting Information) shows complete results for the 2151 always-expressed genes.

### Combining information on age at draw and survival after draw

The results shown in Tables 1 and 2 have limited power relative to the large number of comparisons because expression data were available for only 104 grandparent samples. However, both the association of age with expression level, and the association of expression level with survival after blood draw, contains distinct and important information about the relationship of gene expression to aging in humans. We used two strategies to combine these pieces of information (see Methods, below): (i) a test of the joint null hypothesis that expression is related to neither age nor survival; and (ii) a two-stage model that constructs a multivariate estimator of biological age, then uses it to predict survival. We describe these results below.

### Tests of the joint hypotheses that expression is related to neither age nor survival

**Fig. 1:**
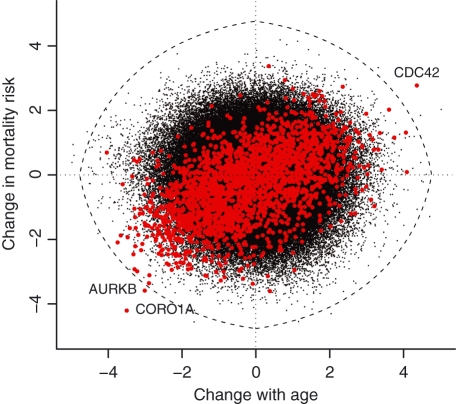
The standardized linear effect of age on each of 2151 expression levels among 104 CEU grandparents is plotted on the *x*-axis against the standardized effect of each expression level on mortality (log hazard rate ratio). Larger red dots indicate the observed values; smaller black dots represent values generated over 100 of the 1000 random permutations of the phenotypic (age, sex, and survival) data associated with each expression vector. Positive values on the *x*-axis indicate increased expression with increasing age; negative values indicate decreased expression with increasing age. Positive values on the *y*-axis indicate increased mortality risk with increased expression; negative values indicate decreased risk. The dashed line is drawn at the 50th largest χ^2^ value observed in 1000 permutations of 2151 genes.

Figure 1 shows the relationship between *Z*-scores for age effects and survival effects on (age-adjusted) expression levels, for the CEU grandparents. Using Fisher’s likelihood ratio approach (Fisher, 1932), we compare the observed *Z*-scores (large red dots) to those generated by a 10% sample of 1000 random permutations of the phenotypic (age, sex, and survival) data, while keeping the expression vectors constant (small black dots). The dashed ellipse is drawn at the 50th largest chi-squared value observed for the 2151 always-expressed genes and 1000 permutations. The results are shown this way to approximate the fifth percentile of the null distribution, adjusted for 2151 comparisons. Three genes fall outside the threshold: CORO1A (0%), CDC42 (0.2%), and AURKB (aurora kinase B; 1.5%). Expression of CDC42 increases with age among the CEU grandparents, and higher age-adjusted expression is associated with higher mortality. CORO1A and AURKB represent the opposite extreme of this same pattern: expression decreases with age, and higher age-adjusted expression is associated with lower mortality.

Overall there is a fairly strong positive correlation (*r* = 0.51; *P*< 2.2 × 10^−16^) between *Z*-scores for age-related expression change and age-adjusted survival in Fig. 1. The orientation of this general pattern is described by the contrast between CORO1A in the lower left quadrant, and CDC42 in the upper right quadrant. Points located in the lower left quadrant (e.g. CORO1A) represent expression levels that decrease with age, and where relative underexpression is associated with higher mortality. Points located in the upper right quadrant (e.g. CDC42) represent expression levels that increase with age, and where relative overexpression is also associated with higher mortality. In contrast, the randomly permuted data are distributed roughly equally around the origin, including many points in the upper left and lower right quadrants. The observed distribution includes relatively few points in these quadrants, and none near the extremes of the null distribution.

It is likely that the results shown in Fig. 1 do not fully illuminate all the patterns of age-related changes in gene expression and survival. Given that cross-sectional data were used to estimate longitudinal patterns of gene expression, we might worry that particular age-related trends in expression are confounded by differential survival, which could cause expressions that do not change with age to appear to do so, or mask true patterns of change. Associations of this type should occur in the upper left or lower right quadrants of Fig. 1, because a positive relationship between expression and mortality should be associated with apparently declining expression, while a negative relationship should be associated with apparently increasing expression.

It is also possible to imagine examples of upper left and lower right quadrant effects with a biological basis. For example, expressions that reflect damage repair or detoxification processes might rise with age and overexpression of these genes might be beneficial to longevity. However, our results offer no persuasive evidence that such associations are very common.

### Multivariate models of biological age vs. survival

It is perhaps intuitively less appealing to examine the association of variation in gene expression for complex traits one gene at a time, than to allow all measured genes to compete with one another simultaneously for inclusion in a model that adjusts for correlations among expression levels. If the number of samples were larger than the number of genes considered, standard multiple linear regression methods might be a suitable approach to this problem. However, like most studies involving microarray data, we have many more potential predictor variables than independent samples. Numerous approaches to the problem of more dimensions than data points have been proposed, but many of them sacrifice interpretability because they collapse correlated expression patterns into composite variables without regard to biological plausibility. Penalized regression approaches can be interpreted much the same as multiple linear regression because they preserve the original data; overfitting is avoided by incorporating a ‘regularization parameter’ that penalizes the likelihood function according to the number of terms in the model. Several different penalized regression schemes have been proposed, including ridge regression (Hoerl & Kennard, 2000), bridge regression (Frank & Friedman, 1993), and the LARS/LASSO (for least angle regression/least absolute shrinkage and selection operator) family of approaches developed by Efron *et al.* (2004).

**Fig. 2:**
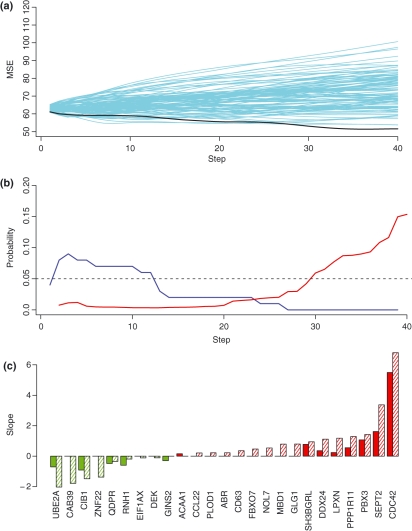
Least absolute shrinkage and selection operator model of biological age. At each step, an additional gene may be added to or subtracted from the model. (a) Mean square classification error (MSE) estimated by cross validation, for the observed data (black line), and 100 random permutations of phenotype data (blue lines); (b) probability of observing MSE less than or equal to the observed MSE (blue), and *P*-value of biological age estimate as a predictor of mortality (red); dashed line is 0.05; (c) slope estimates for gene expressions included in the 14-step model (solid bars), and the 28-step model (hashed bars), showing how the estimated effect of a gene changes as more genes are added to the model. All models between steps 14 and 28 are both significantly better at estimating biological age than models based on random data and significantly better at predicting future mortality than models based on age and sex alone.

In the Methods section, below, we describe estimation and cross-validation procedures for the LASSO model of biological age. Figure 2a shows the leave-one-out cross-validation (LOOCV) curve observed over the first 40 steps (black line), compared to LOOCV curves generated under 100 random permutations of the phenotypic data. In Fig. 2b the blue line plots the probability, at each step, that a model generated from random data has a mean-squared error (MSE) as low as the observed model; the red line plots *P*-values generated by proportional hazards regression using the biological age estimate as a predictor of mortality. It is clear from Fig. 2a,b that the observed LOOCV curve is below the fifth percentile of the distribution of random curves by step 14, and the observed curve remains lower than any randomly generated curve for all steps after step 28. Meanwhile, the predicted biological age generated from the observed model is strongly significantly related to survival after blood draw from steps 2 to 30. The estimated MSE at step 14 is 57, corresponding to a prediction error of ± 7.6 years (7.4 years at step 28). Figure 2c shows the slope coefficients at steps 14 and 28.

The most parsimonious model with an MSE lower than 95% of simulations occurs at step 14. Coefficients of the model, in decreasing order of absolute value, are: CDC42 (5.5), SEPT2 (1.6), PBX3 (1.1), CIB1 (−0.91), SH3BGRL (0.77), UBE2A (−0.71), RNH1 (−0.60), PPP1R11 (0.55), QDPR (−0.48), DDX24 (0.36), GINS2 (−0.30), LPXN (0.24), and ACAA1 (0.16). Clearly, the positive association of CDC42 with age at draw dominates this model. This remains true at steps 20, 40, 60, and 80 (data not shown). Expression levels of CDC42, SH3BGRL, QDPR, and RNH1 were also among the ten most strongly associated with age at draw for CEU grandparents in the univariate analysis reported in Table 1. In the LOOCV models, all the selected terms were included over 85% of the time at step 14, with the exception of LPXN (39%), DDX24 (4%), and ACAA1 (0%).

We used the step 14 model from Fig. 2c to generate estimated biological ages for the CEU grandparents, then include them together with chronological age at draw and sex, in a proportional hazards model of survival. As expected, predicted biological age was positively associated with mortality. The hazard rate ratio (an estimate of relative risk) for a single year increase in estimated biological age was 1.33 (95% CI: 1.10–1.62).

An alternative approach to modeling survival as a function of estimated biological age would be to model it as a function of the difference between biological and chronological age. This is equivalent to forcing both biological age and chronological age to have the same slope (with opposite signs), and is efficient only if both variables are scaled identically. We evaluated this approach by rescaling biological age to have 0 mean and unit variance, then multiplying by the standard deviation of chronological age (7.9) and adding the mean chronological age (71.5); that technique produced results (not shown here) essentially identical to our original method, reported above.

### Multivariate models of survival vs. expression level

The above analysis demonstrates that biological age, estimated from gene expression levels that change with age, is a significant predictor of remaining life span. An important potential weakness of this analysis is that it places too much emphasis on gene expressions that vary systematically with age in cross-sectional data. As noted above, the confounding of differential survival effects with age-related changes in expression could mask the effects of genes that regulate or contribute to survival.

**Fig. 3:**
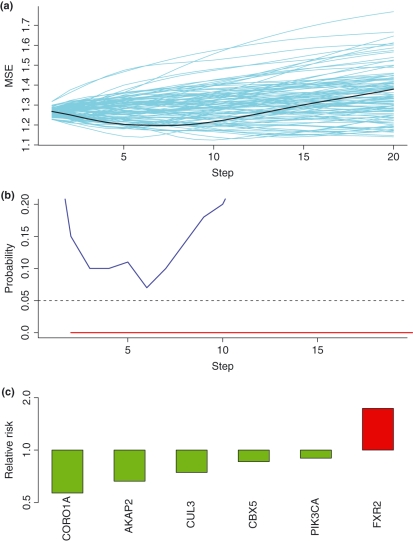
Least absolute shrinkage and selection operator model of survival. At each step, an additional gene is added to or subtracted from the model. (a) Mean square classification error (MSE) estimated by cross validation, for the observed data (black line), and 100 random permutations of phenotype data (blue lines); (b) probability of observing MSE less than or equal to the observed MSE (blue), and *P*-value of overall model as a predictor of mortality (red); dashed line is 0.05; (c) estimated interquartile relative risks for terms included at step 7, showing the estimated effect of a typical variation in gene expression on the relative risk of dying. Models with between four and eight genes included are better at predicting future mortality than 90% of models generated from random data, with a minimum *P*-value of 0.06 at step 7.

In an effort to circumvent this limitation, we also applied the LASSO approach to model survival as a direct multivariate function of expression levels. We computed deviance residuals from a baseline survival model adjusted for age at draw and sex, and used LASSO to identify expression levels associated with variation in the deviance residual [see Methods and reference (Segal, 2006)]. We used the same permuted LOOCV approach for cross validation and permutation tests of significance (described above and in Methods). Results are shown in Fig. 3.

Figure 3a shows the cross-validation results compared to results of 100 random permutations of the phenotype data; a minimum is reached at step 7. In Fig. 3b, the observed cross-validation MSE was smaller than 94% of those observed in permuted data at step 7. Model coefficients are in order of decreasing absolute value: CORO1A (−0.27), FXR2 (0.21), CBX5 (−0.074), PIK3CA (−0.0094), AKAP2 (−0.0086), and CUL3 (−0.0081). The model is dominated by the positive association between FXR2 expression and mortality, and the negative association between CORO1A expression and mortality. Negative associations of AKAP2, CUL3, CBX5, and PIK3CA with mortality also contribute to the model. Table 3 shows that the linear predictors generated by this model are strongly associated with survival: *P*-value = 4.0 × 10^−8^; inter-quartile relative risk (IQRR) = 2.35; median estimated survival difference = 5.5 years. Predicted mortality from the model accounts for 23% of the variation (*R*^2^ = 0.23) in survival among the CEU grandparents. Model coefficients for individual genes are converted into IQRR in Fig. 3c and plotted on a log scale.

**Table 3:** Performance of LASSO mortality model by sex, cause of death, and time since blood draw

Subset	At risk	Deaths	IQRR	*Z*	*P*-value	Median survival 1st quartile	Median survival 4th quartile
All	104	72	2.35	5.49	4.0E−08	89.3	83.8
Males	52	40	2.73	3.76	0.00017	90.5	81.7
Females	52	32	2.31	3.99	6.6E−05	88.8	84.7
Cause of death							
Heart	104	19	2.17	2.65	0.0080		
Cancer	104	14	2.37	2.14	0.032		
Stroke	104	11	3.73	3.54	0.00040		
Diabetes	104	5	7.72	3.21	0.0013		
Inf/Pneu	104	5	3.48	2.49	0.013		
Cognitive	104	10	2.57	2.12	0.034		
Years after blood draw							
1	103	71	2.35	5.46	4.8E−08	89.1	82.6
3	95	64	2.27	4.75	2.0E−06	89.4	84.6
5	88	57	2.53	4.27	2.0E−05	90.4	85.2
10	72	41	2.41	3.30	0.00097	91.5	88.1

IQRR, relative risk comparing the 75th percentile of estimated risk (0.21) to the 25th percentile (−0.03), adjusted for actual age and sex; median survival 1st quartile, estimated median survival for subjects at the 25th percentile of estimated risk (adjusted for age and sex); median survival 4th quartile, estimated median survival for subjects at the 75th percentile of estimated risk (adjusted for age and sex).

The nominal *P*-value given in Table 3 for the overall data is very low, but unsurprising, given that the same survival data were used for estimation and testing. Evaluating the ability to predict survival in selected subgroups (e.g. males vs. females, for various causes of death, or varying numbers of years after blood draw) may be more informative than showing how well the model fits the overall data. Table 3 shows that the model predicts similar mortality risks for males and females. As age is the single largest risk factor for multiple life-threatening diseases, a biomarker that truly reflects biological age (or rate of aging) should be associated with risks of dying from not one, but several common causes of death due to age-related diseases. Therefore, we tested the panel of gene expressions from the survival model (LASSO) for associations with mortality risks for the common causes of death. Table 3 shows that the LASSO model predicts risk from multiple causes of death, in spite of very small sample sizes, with a particularly strong (but imprecisely estimated) effect for deaths attributed to diabetes. It is worth noting that the number of causes of death listed in Table 3 (6) is larger than the number of genes contributing importantly to the model (3), so the possibility that these associations are caused by overfitting seems slight.

Table 3 also shows that the LASSO model remains strongly predictive of mortality for at least 10 years following blood draw. Thus, these associations are not likely due to the presence of terminal diseases in some research subjects at the time of enrollment in the study.

Although Table 3 demonstrates that the predicted model is not strongly affected by subgroup influences on gene expression, a more robust assessment of whether the fit of the model was produced by chance is given by the permutation distribution of cross-validation curves shown in Fig. 3b. The minimum cross-validation MSE of the model is smaller than 94% of those generated by random permutation of the phenotype data (step 7). Therefore, there is approximately a 6% probability that the LASSO model of survival, based on 2151 measures of gene expression, fits our data this well by chance.

### Environmental exposures

Inter-individual differences in gene expression profiles in the Utah CEU lymphoblastoid cell lines may reflect not only heritable genetic influences, but also environmental exposures experienced at any time prior to blood draw. Therefore, we considered the possibility that gene expressions associated with survival may simply reflect exposures (or nonexposure) to a common toxic agent, such as cigarette smoke. The data available to us do not contain information on environmental exposures; however, affiliation with the Church of Jesus Christ of Latter-day Saints (or LDS church), available from the UPDB, indirectly provides information about exposures that affect mortality risks. Merrill *et al.* (2003) using data from the 1996 statewide Utah Health Status Survey, report that 9.2% of LDS men (vs. 24.5% of non-LDS Utah men) reported being current smokers, while only 4.1% of LDS women reported smoking (vs. 23.1% of non-LDS women). Of the 104 grandparents with expression data who linked to the UPDB, 77 (74%) were strongly affiliated with the LDS church, and this is probably an underestimate because UPDB data are very incomplete in this regard. We thus would expect only a small number (probably less than ten) of the grandparents to have been smokers. In previous work with the UPDB, we have shown that reduced smoking and alcohol consumption among active church members probably accounts for 1.3 additional years of life expectancy compared to Utahans unaffiliated or inactive in the church (Kerber *et al.*, 2001). Inclusion of church affiliation as a covariate in the survival models slightly strengthens the relationship of the model predictions to survival (data not shown), indicating that the results reported in Table 3 are not confounded by smoking. Furthermore, none of the genes listed in Table 2, or included in the LASSO survival model, have been reported to be significantly affected by cigarette smoking (Ryder *et al.*, 2004; van Leeuwen *et al.*, 2005).

It is apparent in Tables 1 and 2 that spouse correlations for some individual gene expressions are moderately high. Across the entire set of 2151 always-expressed genes, the highest observed spouse correlation is 0.50 (PRSS3). CORO1A and IQGAP1 exhibit spouse correlations > 0.2, although the estimated heritability for each is quite high. The mean spouse *r*^2^ across the entire dataset is 0.962 while the maximum *r*^2^ for 100 random pairs of grandparents was slightly smaller (0.958; minimum = 0.952, mean = 0.955). Overall, then, spouse expression profiles are slightly more strongly correlated than expected by chance, although the level of correlation among all expression profiles is very high. Interestingly, however, the correlation of mortality risk between spouses (using the deviance residuals from the baseline proportional hazards model – see Methods) is only 0.075 (*P*-value 0.45), so correlations in expression are not likely to confound the survival analysis.

## Discussion

It is not surprising to find that gene expression patterns vary with age, nor that variation in gene expression correlates with variation in longevity; many studies in model organisms have clearly demonstrated these associations. However, the observation that patterns of gene expression in lymphoblastoid cell lines contain information about both the age and subsequent survival of human donors is both biologically interesting and potentially of great clinical utility.

While Fig. 1 shows clearly that, in general, the intensity and direction of age-related change in expression of a gene among the CEU grandparents is related to the strength of association of that gene’s expression with survival, identifying individual genes that are most strongly related to aging is less simple. We have taken a variety of approaches to this task, and several genes appear to be important in more than one context. In particular, CDC42 and CORO1A appear to be associated with both age at draw and survival after blood draw, whether univariate or multivariate approaches are applied. CDC42 expression increases with increasing age among the CEU grandparents, and, after adjusting for age, higher expression of CDC42 is associated with higher mortality. CDC42 is also the dominant factor in our multivariate model of biological age, which is a significant predictor of mortality. Recently, Wang and colleagues reported that mice deficient in a negative regulator of Cdc42 (Cdc42GAP) exhibited constitutively elevated Cdc42 expression and phenotypes consistent with premature aging, including: osteoporosis, muscle atrophy, and impaired wound-healing. According to Wang *et al.*:

“Our findings that Cdc42 activation is associated with natural aging and that Cdc42GAP deficiency causes a DNA damage repair defect to allow cells to accumulate genomic abnormalities that leads to early senescence further suggest a functional link between Cdc42 activity and mammalian aging (Wang *et al.*, 2007).”

The results of this study support this conclusion.

Coronin (*CORO1A*) is an actin-binding protein with potentially important functions in both T-cell mediated immunity and mitochondrial apoptosis (Dustin, 2006; Foger *et al.*, 2006; Mueller *et al.*, 2008). Shiow *et al.* (2008) report that coronin defects in mice cause peripheral T-cell deficiency, and describe a human patient with severe combined immunodeficiency who had mutations in both coronin alleles. A nonsense mutation in CORO1A has recently been shown to suppress autoimmune response in a mouse model of systemic lupus erythematosus (Haraldsson *et al.*, 2008), further suggesting that coronin is critical to immune functioning. Moreover, the inadvertent coronin knockout mice of Haraldsson *et al.* show substantially decreased mitochondrial membrane potential and increased apoptosis in T cells, but not in B cells.

Several other genes exhibiting associations with either aging or survival are worthy of note. AURKB is a key member of the chromosomal passenger complex which is critical in the regulation and conduct of mitosis (Ruchaud *et al.*, 2007). Inhibition of AURKB in tumor cells leads to growth inhibition and apoptosis (Yang *et al.*, 2007). CBX5 encodes the human HP1α heterochromatin protein, importantly involved in the construction and maintenance of chromatin and hence an important regulator of gene expression. CBX5 expression decreases with age in the CEU grandparents, and reduced expression is associated with greater mortality. Likewise, reduced expression of IQGAP1 is associated with increased mortality, although expression of IQGAP1 is not strongly related to age. Interestingly, IQGAP1 is an effector of CDC42, and is involved in multiple signaling pathways (Brown & Sacks, 2006). Goring *et al.* (2007) report a LOD score for cis-regulation of IQGAP1 expression of 5.8 (chromosome 15, 99 cM). Similarly, we find that, in the 60 CEU grandparents genotyped by the International HapMap Projects (http://www.hapmap.org), several single nucleotide polymorphisms near the IQGAP1 gene are strongly associated with IQGAP1 expression (rs1702161, rs3862432, rs3862435, and rs3862436).Thus, the region immediately surrounding IQGAP1 may harbor genetic variants associated with variation in human lifespan.

It is puzzling that relative overexpression of TERF2 interacting protein (TERF2IP, aka hRAP1) in lymphoblastoid cell lines is associated with increased mortality, because increased expression of TERF2IP should lead to increased telomere length (Li & de Lange, 2003), which has been associated with decreased mortality in the CEU grandparents (Cawthon *et al.*, 2003). Unlike IQGAP1, variation in TERF2IP expression is not highly heritable, either in transformed (*H*^2^ = 0.09; *P*= 0.10 in our data) or untransformed (*H*^2^ = 0.15; *P*= 0.054 in Goring *et al.*, 2007) lymphocytes. In fact, TERF2IP expression was uncorrelated with subjects’ telomere lengths as measured in whole blood (*r* = 0.04). This may be a consequence of the cell transformation process, which activates telomerase so that cell lines may grow indefinitely in culture; possibly variable TERF2IP expression is marking some variation in telomerase activity in transformed lymphocytes that is indirectly related to longevity. A recent report links longer telomeres to increased risk of breast cancer (Svenson *et al.*, 2008). Among the CEU grandparents, however, TERF2IP expression was not significantly associated with cancer mortality risk.

Because of the large number of comparisons we have generated for a relatively small number of samples, and because the potential for measurement error is inherent in microarray data, the associations we have observed require confirmation in other settings. Associations between gene expressions and survival may be due to causal roles in regulating aging; alternatively, the expression of a gene may not reflect a regulatory role in aging, but nevertheless may serve as a reliable indicator of overall health and remaining life expectancy. Strong evidence for a regulatory role in aging would be provided by experiments in model systems showing that modulating expression extended lifespan. Within the CEU family data, we are pursuing linkage and genetic association studies to identify loci responsible for regulating expression of the heritable expression phenotypes associated with longevity. The identification of germ line variants that affect the expression of longevity-associated genes will facilitate further high-throughput genetic epidemiologic studies of human aging. However, even the nonheritable gene expression patterns we have reported may have considerable relevance to mechanisms of aging and age-related disease processes. Finally, we note that expression data are available for approximately 6000 genes not included in the ‘always-expressed’ list to which we restricted this analysis. Approximately half of these genes are expressed in many, but not all samples. The other half are rarely, if ever, expressed above background. We plan to investigate the association between longevity and expression of these genes as well.

We have described some striking patterns of association of gene expression with age and mortality, based on lymphoblastoid cell lines derived from ordinary blood samples, and stored for years as a replenishable source of DNA for genetic studies. If these results can be confirmed by others, frozen cell lines may also have considerable value as sources of phenotypic information on transcription, translation, and other cellular processes helpful in predicting the future health of the donors.

## Materials and methods

The CEPH/Utah family resource originated from bloods drawn from 46 three-generation families, each consisting of 5–15 siblings, their two parents, and two to four grandparents who were still alive at the time of the family blood draws in the early 1980s (White *et al.*, 1985; Dausset *et al.*, 1990). All subjects signed written forms giving informed consent for samples to be used in health research on the condition that names were not to be released in reports or published. The University of Utah IRB has approved this research project.

Cheung and co-workers (Cheung *et al.*, 2003, 2005; Morley *et al.*, 2004) extracted RNA from transformed B lymphocytes obtained from the Coriell Cell Repository (http://locus.umdnj.edu/nigms/ceph/ceph.html) for a total of 247 CEU family members (124 male; 123 female), including 104 of the grandparents for whom we had survival data. Expression levels for 8793 probesets were measured using Affymetrix HG-Focus arrays. The resulting expression data were deposited in the NCBI Gene Expression Omnibus database, accession numbers GSE1485 and GSE2552. We combined both datasets to maximize the number of individuals available for study.

Each probeset on the HG-Focus array consists of a set 11–20 pairs of probes, each consisting of a 25-mer oligonucleotide representing a ‘perfect match’ to the target sequence and a ‘mismatch’ probe made by substituting an alternative nucleotide at the 13th position. Several recent studies (Mecham *et al.*, 2004; Dai *et al.*, 2005; Liu *et al.*, 2007) have shown that the original mapping of probes and probesets to genes, based on the human UniGene Build 133 database, contains many errors. To improve the fit of probesets to genes, we used the ‘HG-Focus RefSeq Transcript’ mapping supplied by Liu *et al.*, available from http://gauss.dbb.georgetown.edu/liblab/affyprobeminer/transcript.html. After re-mapping, 8174 probesets were available for analysis.

We tested whether each gene was expressed beyond baseline in each sample using the Wilcoxon signed rank test as described in the Affymetrix Microarray Suite version 5 (Liu *et al.*, 2002). This is a test for absolute ‘presence’ vs. ‘absence’, testing if the observed signals for each probeset were significantly greater than background. For purposes of the present analysis, we eliminated from consideration all probesets that were not called ‘present’ (*P*< 0.04) or ‘marginal’ (*P*< 0.06) in each of the grandparents’ samples. This step left us with 2151 always-expressed genes.

We used Wu and Irizarry’s GeneChip robust multiarray averaging (gcrma) method to normalize all expression levels(Wu & Irizarry, 2005). The gcrma and mas 5.0 algorithms we employed were implemented in the ‘affy’ and ‘gcrma’ packages available from the Bioconductor website (http://www.bioconductor.org).

We evaluated all samples for outlying observations in relation to average background, scale factor, number of genes called ‘present’, and 3′ to 5′ ratios for GAPDH, following the procedures described by Wilson *et al.* (2004). We excluded 12 samples from analysis because they were out-of-range for at least one test. In addition, five samples exhibited inappropriately high or low levels of expression of both RPS4Y1, encoded on the Y chromosome, and the X-inactivating sequence transcript (XIST), expressed only in women. Without exception, in women high expression of RPS4Y1 was coupled with low expression of XIST, and in men low expression of RPS4Y1 was coupled with high expression of XIST. As all grandparents are by definition fertile, we could rule out sex chromosome abnormalities as an explanation. We excluded these samples on the grounds that they had been mistakenly attributed to the wrong person. After these exclusions, at least one sample from 238 individuals (including all 104 grandparents) remained.

Many CEU family members, including most of the grandparents, had expression data available from multiple arrays (usually two, although two of the grandparents had four arrays available). For these individuals, we averaged gcrma-corrected expression levels for each probeset prior to analysis.

### Univariate analyses

We performed two separate analyses of expression as a function of age at draw. First, we considered only the grandparents, who were effectively unrelated to one another, although careful analysis of the records of the UPDB (Wylie & Mineau, 2003) has revealed that a few of the CEU grandparents are distantly related. The grandparents’ expression data represented were treated as independent observations, and we used ordinary least squares methods to regress expression level for each of the always-expressed probesets against age, adjusting for sex. Zhang *et al.* (2007) have already reported on patterns of sex-specific expression in these data.

Strong genetic correlations in expression level should be present within each three-generation CEU family. For this reason, standard linear regression approaches are not appropriate for the study of age-related variation in expression patterns. Instead, we used linear mixed-effects models to adjust for the kinship among family members (Therneau, 2007). The substantially larger sample size (238 vs. 104) and wider range of ages at draw (5–97 vs. 57–97) led us to consider both linear and quadratic effects for age at draw in the three-generation families. Otherwise, the model fit was the same as for the grandparents: expression level was modeled as a function of age at draw, age squared, and sex. Heritability estimates were computed for three-generation pedigrees using SOLAR (Almasy & Blangero, 1998). These results are given in TablesS1 and S2 (Supporting Information).

### Survival models

Grandparents ranged in age from 57 to 97 years of age at the time of the blood draw. Median follow-up age was 84.7 years (range 65.7–100.8). Survival was measured from age at draw to age at death or follow-up. We used a proportional hazards model adjusting for sex, year of birth, and age at draw to test the association of each expression level with survival. Each proportional hazards model was tested for nonproportionality using the cox.zph function in the r software package (http://www.r-project.org). Although some nonproportionality was detected with *P*-values below 0.0001, none of the genes strongly associated with survival had a *P*-value for nonproportionality lower than 0.28 (EMP3).

### Bivariate age-at-draw vs. survival models

We computed Fisher’s (1932) likelihood ratio test of the composite null hypothesis that expression was unrelated to either aging or longevity. We generated 1000 random permutations of the survival data because of concerns that age at blood draw was not completely independent of survival after blood draw, even under the null hypothesis. Random permutations were generated by shuffling the rows of a matrix that included age at draw, age at follow-up, sex, and vital status as columns. For each iteration, the randomly ordered phenotypes were assigned to the unpermuted gene expression vectors, and computed the linear regressions on age at draw, the proportional hazards models and the Fisher test. This procedure allowed the correlation structure of the expression data, and the correlation structure of the survival data to remain intact, while testing the relationship between the two datasets.

### Adjustment for multiple comparisons

Adjustments for multiple testing are clearly appropriate in this context, but many methods mask hidden assumptions about the dependency structure of the data or the true proportion of false null hypotheses (Reiner *et al.*, 2003). Even permutation-based methods may retain some vulnerability to hidden dependencies within microarray data (Efron & Tibshirani, 2007). We therefore employ a simple Bonferroni correction in presenting the results of the univariate analyses, and a Monte Carlo permutation test in presenting the results of the bivariate analyses.

### Multivariate analyses

#### LASSO models of biological age

To assess the relationship between age at draw and multiple expression levels, we employed the LASSO algorithm of Tibshirani (Tibshirani, 1996; Efron *et al.*, 2004) to build a linear model of age at draw as a function of multiple expression levels. We used only the grandparents’ data to avoid complex dependency structures. Briefly, the LASSO approach minimizes the residual sum of squares in a multiple regression model subject to the constraint that the sum of the absolute values of the standardized coefficients is less than a specified constant. Efron *et al.* (2004) showed that computing all possible LASSO models is feasible and provides a basis for rationally choosing among them, by minimizing *C*_p_ or by cross validation.

Cross-validation procedures divide the data at random into *K* equal subsets, and, for *i*=1 to *K*, use all the data not in the *i*th subset to estimate the model, and the data in *i* th subset to test the model predictions. The goal is to find the value of the tuning parameter that minimizes the mean square prediction error across the *K* subsets. With relatively small datasets, however, *K*-fold cross-validation procedures are often unstable (Segal, 2006) – this proved to be the case with the present data set.

We set *K*=104, which leads to the LOOCV procedure (LOOCV) in our sample of 104 grandparents. We compared the resulting LOOCV curve to curves generated from 100 random permutations of the data, performed as described above. Comparing the cross-validation curve to a null distribution of cross-validation curves not only gives information on the optimal setting of the tuning parameter, but also on the probability that the result is due to chance.

For each value of the tuning parameter, corresponding to a step in which a predictor variable can be added or dropped, the model selected was used to predict each subject’s age at draw. These ‘biological age’ estimates, together with the subjects’ actual age and sex, were used to predict age at death in a proportional hazards model.

#### LASSO models of survival after blood draw

We followed the approach of Segal (2006), using the LASSO approach described above to regress the deviance residuals of a baseline proportional hazards regression (adjusted for sex and age at draw) against the set of expression levels. These permuted LOOCV approach described above to identify optimal settings for the tuning parameter and to assess the probability that the observed pattern was the result of chance.

## Supporting Information

Fig. S1 Illustrations of shape categories defined in Table S1.

Table S1 Categories of age-related changes in expression level observed in 2151 always-expressed genes in three-generation CEU family data.

Table S2 Top 20 age-associated expression levels in threegeneration families.

Table S3 Complete list of age and mortality associations for 2151 always-expressed genes.
