# Supplementary material for: Gene expression profiles associated with aging and mortality in humans
Source: Aging Cell. 2009 Jun;8(3):239–50. doi: 10.1111/j.1474-9726.2009.00467.x (PMC2759984; doi:10.1111/j.1474-9726.2009.00467.x)
Supplement: Supplementary file 1 [file ace0008-0239-SD1.doc]

**Supplementary Material (to be accessed on-line)**

*Changes in gene expression with age: three-generation families*

We modeled expression as a simple linear function of age at draw using all three generations of the CEU families. Out of the full set of 2,151 always-expressed genes, 784 (36.4%) expression levels showed age effects with p-values below the Bonferroni 5% threshold of 2.3 x 10^-5^. Of these, 348 increased with age, and 436 decreased with age. A larger number of age-related changes were observed when we added a quadratic term to the model, allowing for curvature in the regression of expression against age. A two degree-of-freedom test of significance of the combined linear and quadratic effects yielded 907 (42.2%) expression levels significantly associated with age at draw, allowing for multiple comparisons.

For comparative purposes, we classified the shape of the relationship between age at draw and expression level into nine categories, labeled A-I for convenience. The category definitions are listed in Supplementary Table 1 and idealized representations of each are displayed in Supplementary Figure 1. More than half (1244; 57.8%) of the expression levels were not associated with age strongly enough to overcome the Bonferroni adjustment; of these, 443 (20.6%) exhibited no significant association with age at draw even at a nominal p-value of 0.05. Categories A (superlinear rise) and I (superlinear drop) had no members with p-values exceeding the Bonferroni threshold. The quadratic-only categories D (U-shaped) and F (inverted U) were rarely observed, with only 13 and 8 members, respectively. We note that the expression levels are reported on a log2 scale; hence, a linear increase (B) or decrease (H) in measured expression level corresponds to a multiplicative increase in gene expression, while a truly linear change in gene expression corresponds to a sublinear change (C or G) on a log scale. Supplementary Table 3 lists individual results for all 2,151 always-expressed genes.

Supplementary Table 2 lists the top 20 associations in the three-generation families in increasing order of p-value. Note that 17 of the 20 strongest associations in Table 2, are negative overall (i.e., the regression slope of a model that omits the age^2^ term is negative), and the shape category is either G (sublinear drop) or H (linear drop). Expression of SAFB and PSMD4 increases sublinearly with age, and expression of BAT2 increases linearly.

## Figure 1

**Supplementary Figure 1. Illustrations of shape categories defined in Supplementary Table 1.**

## Supplementary Table 1. Categories of age-related changes in expression level observed in 2,151 always-expressed genes in three-generation CEU family data.

| **Category** | **Shape** | **Linear Effect** | **Quadratic Effect** | **Count** | **Percent** |
| --- | --- | --- | --- | --- | --- |
| Superlinear Rise | A | Positive | Positive | 0 | 0.0% |
| Linear Rise | B | Positive | Nonsignificant | 148 | 6.9% |
| Sublinear Rise | C | Positive | Negative | 257 | 11.9% |
| U-shaped | D | Nonsignificant | Positive | 13 | 0.6% |
| Unrelated to Age | E | Nonsignificant | Nonsignificant | 1244 | 57.8% |
| Inverted U | F | Nonsignificant | Negative | 8 | 0.4% |
| Sublinear Drop | G | Negative | Positive | 232 | 10.8% |
| Linear Drop | H | Negative | Nonsignificant | 249 | 11.6% |
| Superlinear Drop | I | Negative | Negative | 0 | 0.0% |

**Supplementary Table 2. Top twenty age-associated expression levels in three-generation families.**

| **Probeset** | **Z.lin** | **Z.age** | **Z.age^2^** | **Z.sex** | **Shape** | **p-value** | **Gene Symbol(s)** |
| --- | --- | --- | --- | --- | --- | --- | --- |
| HG-FocusHF4679 | -15.14 | -7.83 | 4.22 | 1.42 | G | 2.47E-39 | PRKAR1A |
| HG-FocusHF9208 | -15.46 | -5.51 | 2.08 | 0.92 | G | 6.58E-38 | EIF3S10 |
| HG-FocusHF5720 | -14.27 | -7.27 | 4.27 | 0.71 | G | 1.25E-36 | SF3B1 |
| HG-FocusHF9350 | 14.53 | 7.51 | -3.97 | -0.93 | C | 1.85E-36 | SAFB |
| HG-FocusHF5595 | -14.31 | -6.78 | 3.49 | 2.23 | G | 1.27E-35 | RNF11 |
| HG-FocusHF9502 | -13.90 | -7.45 | 3.94 | -0.90 | G | 1.78E-34 | IFNA1, IFNA2, IFNA4, IFNA6, IFNA7, IFNA10, IFNA13, IFNA14, IFNA16, IFNA17 |
| HG-FocusHF5779 | -13.24 | -7.55 | 4.39 | -1.46 | G | 3.87E-33 | BCL10 |
| HG-FocusHF8192 | -12.65 | -8.40 | 5.40 | 0.55 | G | 4.88E-33 | MARCH7 |
| HG-FocusHF8737 | -11.45 | -9.90 | 7.01 | 0.18 | G | 1.69E-32 | SH3BGRL |
| HG-FocusHF3239 | 13.94 | 4.79 | -1.61 | -1.19 | B | 2.07E-32 | BAT2 |
| HG-FocusHF4119 | 13.04 | 6.76 | -3.92 | 0.15 | C | 2.55E-31 | PSMD4, PSMD4P2 |
| HG-FocusHF10285 | -13.52 | -4.71 | 1.56 | 1.28 | H | 9.53E-31 | SEC24B |
| HG-FocusHF1562 | -12.56 | -6.69 | 3.92 | 1.03 | G | 2.21E-30 | TANK |
| HG-FocusHF10040 | -13.81 | -1.71 | -1.25 | 0.48 | H | 6.67E-30 | SFRS2IP |
| HG-FocusHF1673 | -13.22 | -4.23 | 1.29 | 0.81 | H | 8.14E-30 | SMNDC1 |
| HG-FocusHF2428 | -12.83 | -5.85 | 2.85 | 0.70 | G | 8.28E-30 | MARCKS |
| HG-FocusHF1383 | -12.91 | -5.76 | 2.95 | 0.71 | G | 8.97E-30 | AGL |
| HG-FocusHF6922 | -11.40 | -8.35 | 5.81 | 0.99 | G | 1.74E-29 | HNRPH1 |
| HG-FocusHF1269 | -13.10 | -3.58 | 0.66 | 1.95 | H | 3.82E-29 | C1D |
| HG-FocusHF2499 | -12.70 | -5.18 | 2.34 | 0.67 | G | 7.27E-29 | VPS4B |

Notes: Probeset—probeset name from HG-Focus Refseq transcript library(see reference (Liu et al., 2007)); Z.lin—Z score of linear model of expression vs. age, adjusted for sex; Z.age—Z score of linear term of linear + quadratic model of expression vs. age, adjusted for sex; Z.age^2^—Z score of quadratic term in linear + quadratic model; Shape—relationship between age and expression, as defined in Table 1 and Figure 1; p-value—probability of observing a χ^2^ [2 d.f.] greater than the observed likelihood ratio test for the linear + quadratic model of expression vs. age; Gene Symbol(s)—HUGO symbol name or names corresponding to current mapping of probeset sequence.

## In general, the pattern of age-related changes observed among the grandparents alone, reported in Table 3 and Supplementary Table 3, is quite different from the pattern observed across all three generations. The correlation coefficient between the linear-only Z score for three generations and the linear Z score for grandparents-only is -0.09. This is to be expected if many age-related shifts in gene expression across the three generations are linked to the developmental changes that occur during growth and maturation.
